# An Internally Quenched Fluorescent Peptide Substrate for Protealysin

**DOI:** 10.1038/s41598-019-50764-2

**Published:** 2019-10-04

**Authors:** Maria A. Karaseva, Ksenia N. Chukhontseva, Irina S. Lemeskina, Marina L. Pridatchenko, Sergey V. Kostrov, Ilya V. Demidyuk

**Affiliations:** 1 Institute of Molecular Genetics, Russian Academy of Sciences, Moscow, Russia; 2 V.L. Talrose Institute for Energy Problems of Chemical Physics, N.N. Semenov Federal Research Center of Chemical Physics, Russian Academy of Sciences, Moscow, Russia

**Keywords:** Proteases, Proteases

## Abstract

Protealysin, a metalloprotease of *Serratia proteamaculans*, is the prototype of a subgroup of the M4 peptidase family. Protealysin-like proteases (PLPs) are widely spread in bacteria but also occur in fungi and certain archaea. The interest in PLPs is primarily due to their putative involvement in the bacterial pathogenesis in animals and plants. Studying PLPs requires an efficient quantitative assay for their activity; however, no such assay has been reported so far. Here, we used the autoprocessing site sequence of the protealysin precursor to construct an internally quenched fluorescent peptide substrate 2-aminobenzoyl-L-arginyl-L-seryl-L-valyl-L-isoleucyl-L-(ε-2,4-dinitrophenyl)lysine. Protealysin and thermolysin, the prototype of the M4 family, proved to hydrolyze only the Ser-Val bond of the substrate. The substrate exhibited a K_M_ = 35 ± 4 μM and k_cat_ = 21 ± 1 s^−1^ for protealysin as well as a K_M_ = 33 ± 8 μM and k_cat_ = 7 ± 1 s^−1^ for thermolysin at 37 °C. Comparison of the effect of different enzymes (thermolysin, trypsin, chymotrypsin, savinase, and pronase E) on the substrate has demonstrated that it is not strictly specific for protealysin; however, this enzyme has higher molar activity even compared to the closely related thermolysin. Thus, the proposed substrate can be advantageous for quantitative studies of protealysin as well as for activity assays of other M4 peptidases.

## Introduction

Protealysin (PLN), a metalloprotease of *Serratia proteamaculans*, is the prototype of a subgroup of the M4 peptidase family (https://www.ebi.ac.uk/merops/cgi-bin/famsum?family=M4)^1^. Protealysin-like proteases (PLPs) are widely spread in bacteria but also occur in fungi and certain archaea. The interest in PLPs is primarily due to their probable involvement in the bacterial pathogenesis in animals and plants. For instance, PLN and grimelysin of *Serratia grimesii*^2^ were shown to mediate bacterial invasion of eukaryotic cells^3–6^; protease Zpx of *Cronobacter turicensis*, a causative agent of life-threatening systemic infections in premature, low-birth-weight, and immune-compromised neonates is presumably a component of the bacterial mechanism of immune evasion^7^; protease PrtS of the insect-pathogenic bacterium *Photorhabdus luminescens* can suppress the antibacterial defense in insects^8,9^; while protease Prt1 of the plant-pathogenic bacterium *Pectobacterium carotovorum* affects extensins, plant cell wall protein, which makes Prt1 a putative factor of bacterial penetration into plant cells^10^.

Currently, the activity of PLPs is assayed using protein substrates, most commonly, casein (typically azocasein)^8–16^ or less commonly actin^3,15,17^. The applicability of these substrates is somewhat limited. In the case of casein, numerous bonds are cleaved and the enzymatic reaction is evaluated by the products not precipitated by trichloroacetic acid. Thus, the degree of substrate conversion cannot be correctly evaluated. In addition, the kinetics monitoring interferes with the precipitation-based end point assay. Actin is specifically digested by PLPs^3^. However, the routine analysis relies on the fractionation of the hydrolytic products by gel electrophoresis, which makes it time- and labor-consuming. A well-known substrate of neutral proteases N-(3-[2-Furyl]acryloyl)-Gly-Leu amide (FAGLA)^18^ was tested. However, PLN proved to hydrolyze FAGLA with very low efficiency (k_cat_/K_M_ is roughly 100 times lower compared to thermolysin, a member of the same M4 family of peptidases)^12^, which makes it impracticable for PLP activity assay.

The problem of a convenient protease substrate can be successfully obviated by using reporter systems based on Förster resonance energy transfer (FRET). FRET substrates of proteases are peptides corresponding to the enzyme specificity with a donor fluorophore at one end and a fluorescence acceptor at the other. The acceptor can be “dark” and function as a quencher or fluoresce. In addition to the flexibility, FRET substrates are advantageous in high sensitivity of the corresponding methods and the possibility of the continuous kinetic assay. FRET substrates are used in the activity assays for a variety of proteases including certain M4 peptidases^19–25^. However, no FRET substrates have been created for PLPs.

Here, the sequence of the autoprocessing site of the PLN precursor was used to construct an internally quenched fluorescent peptide substrate. The substrate examination has demonstrated that it can be efficiently used for quantitative analysis of PLN catalytic properties and can be also used to assay the activity of other M4 peptidases.

## Results and Discussion

### Substrate design

**Figure 1:**
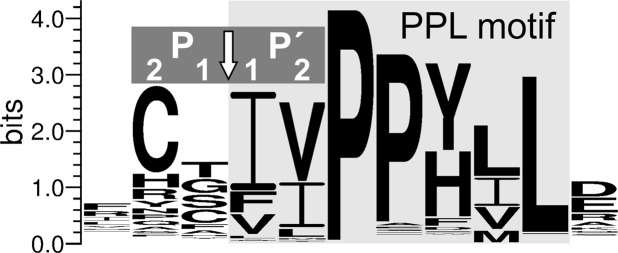
LOGO presentation of processing site and PPL motif in precursors of protealysin-like proteases. The arrow indicates the cleavage site. The processing site residues are designated as P1, P2, P1′, P2′ according to Schechter and Berger^26^. Light gray shading indicates the PPL motif.

The substrate specificity of PLPs has not been studied systematically^1^. At the same time, the analysis of the PLN spatial structure in comparison with that of thermolysin (TLN) indicates that, similar to other M4 peptidases, the S1′ site (as designated by Schechter and Berger^26^) is the major determinant of the substrate specificity of PLPs^27^. However, the interactions at the S2′ site of PLN were proposed to modulate the efficiency of substrate binding more than for TLN. The interactions of the substrate downstream of the scissile bond are determined by the S2 and S1 sites of the enzyme and are largely analogous to those of TLN^27^. Thus, efficient PLN binding requires four substrate sites (P2, P1, P1′, and P2′). The hydrolysis sites have been determined for several proteins by certain PLPs (PLN, grimelysin, and Prt1). These data indicate the preference of the enzymes for hydrophobic residues at the P1′ and P2′ positions, which was more pronounced for the residues with less voluminous side chains compared to TLN. The preference for the P1 and P2 positions was less pronounced; however, a bias to small and polar residues at P1 and charged ones at P2 was observed^1,3,10,17,28,29^. The substrate sequence was selected based on the autocatalytic maturation data for PLP precursors. It was shown that PLN is accumulated in the cell as an inactive precursor, which is processed and activated after cell lysis^29,30^. PLN contributes most to the processing. It goes step-by-step; and the first autoprocessing event, the detachment of seven N-terminal residues, takes place within the cell^29^, and the processing site is located directly upstream of the PPL-motif conserved for propeptides of PLPs (Fig. 1)^1,31^. The substrate design was based on the PLN processing site Arg-Ser−|−Val-Ile (“−|−” is the cleaved bond). At the same time, the residues at the P1′ and P2′ positions of the processing site in PLN are common in other enzymes of the group, suggesting that such substrate can be applied for the activity assay of all PLPs disregarding their nuances in substrate specificity.

Next, we selected the donor fluorophore and fluorescence acceptor. Currently, a variety of donor-acceptor pairs are used in protease substrates^23,25,32,33^. They vary in the structure, spectral and other physicochemical properties, and not least in price. We opted for the long and widely used pair 2-aminobenzoyl/2,4-dinitrophenyl (Abz/Dnp). This pair has a clear drawback relative to newer products, high background fluorescence when used as a part of internally quenched fluorescent substrates^33^. This results in a relatively low sensitivity of activity assays based on Abz/Dnp peptides; however, it is not critical as demonstrated by numerous examples of their successful application to address standard problems^32^. At the same time, the Abz/Dnp pair has clear merits^34^. Abz is the smallest fluorescent group described so far, which ensures the solubility of Abz-containing peptides. Dnp is a stable quencher and its absorption spectrum does not change with pH, which makes this group a convenient marker for substrate quantitation in solutions. Finally, Abz and Dnp are inexpensive, can be readily integrated into peptide substrates, and are among standard modifications provided by most custom peptide synthesis services.

Abz was attached to the N terminus of the peptide. Dnp was introduced into the peptide by linking to the ε-amino group of lysine added to the C terminus of the peptide. Thus, the substrate proposed in this study has the following structure: Abz-Arg-Ser-Val-Ile-Lys(Dnp) (Abz-RSVIK(Dnp)).

### Substrate absorption and solubility

Abz-RSVIK(Dnp) features sorption onto quartz cuvettes for spectrophotometry, microplates for fluorescence-based assays, and microcentrifuge tubes including low protein-binding tubes (supplementary Fig. S1), which has to be accounted for when using this substrate. First, we excluded the common serial dilutions and prepared all working solutions directly from the substrate stock in DMSO. Second, substrate solutions were incubated in plate wells prior to enzymatic assays, which obviated concentration changes during the experiment (Supplementary Fig. S2). Third, the concentration alterations from absorption were controlled in a parallel control experiment without the substrate for each activity assay to evaluate the substrate concentration directly in the reaction mixture. (All details of the assay for the initial hydrolysis rate are described in Methods.) As concerns the sorption onto spectrophotometer cuvettes, it is relatively low (Supplementary Fig. S1A), and considering that obviating it is hardly realistic, we tried to perform the measurements as fast as possible. In our experiments, the optical density was measured 10–15 s after solutions were added to cells.

The substrate sorption on labware complicated the measurement of the Abz-RSVIK(Dnp) solubility since the standard approach based on serial dilutions and identification of the linear range of the experimental concentration-dilution factor relationship could not be used. The substrate solubility was evaluated by periodic spectrophotometric quantitation of its dilutions immediately prepared from the stock in DMSO for one day. Up to 130 µM, the substrate solutions remained stable for 24 h (Supplementary Fig. S3); however, starting from 150 µM their optical density decreased with time. Thus, the substrate has reasonable water solubility and can be used in concentrations up to 130 µM.

### Identification of substrate hydrolysis site by protealysin and thermolysin

**Figure 2:**
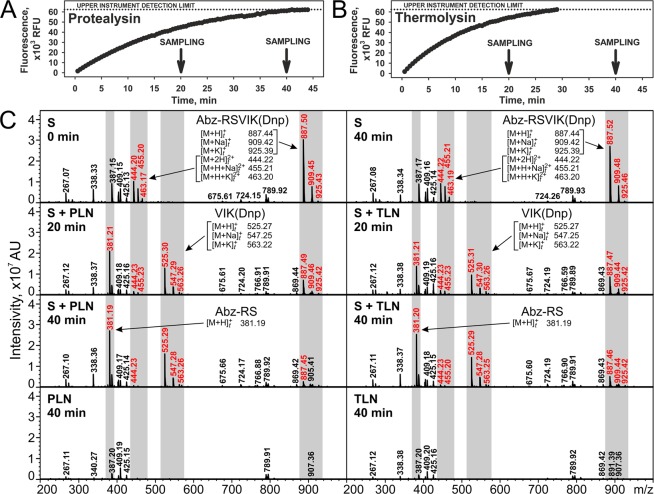
Analysis of Abz-RSVIK(Dnp) cleavage by protealysin (PLN) and thermolysin (TLN). (**A**,**B**) Kinetics of Abz-RSVIK(Dnp) hydrolysis by PLN and TLN, respectively. The arrows indicate time points of mass spectrometry. RFU, relative fluorescence units. (**C**) Mass-spectrometric analysis of reaction mixes. The peaks corresponding to the substrate and hydrolysis products are indicated by red font and gray vertical bars. S, substrate; AU, arbitrary units; m/z, mass-to-charge ratio; [M + nH/K/Na(+Na/K)]^Z+^_t_, theoretical m/z values.

Mass spectrometry of the reaction mixtures was used to identify the site of Abz-RSVIK(Dnp) hydrolysis by PLN and TLN. This analysis detected only the products corresponding to hydrolysis at the Ser-Val bond, which is also cleaved in the natural amino acid sequence reproduced in the synthetic peptide (Fig. 2). The specificity of the enzymes also includes cleavage of the bond preceding Ile suggesting the Val-Ile digestion of the substrate. However, even if such reaction takes place it proceeds substantially slower and its products remain undetectable after the time period (40 min) by far exceeding that required for almost complete peptide hydrolysis at Ser-Val. In the context of Abz-RSVIK(Dnp) application in the activity assays, it is fair to say that its hydrolysis occurs at the only bond. Thus, the quantity of the cleavage product detected by its fluorescence is equivalent to the quantity of hydrolyzed substrate, which allows adequate quantitation of the enzyme kinetics.

### Kinetic parameters for substrate hydrolysis by protealysin and thermolysin

**Figure 3:**
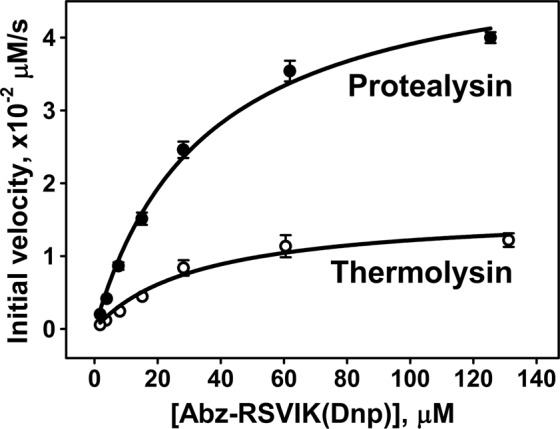
The dependence of the initial rates of Abz-RSVIK(Dnp) hydrolysis by protealysin and thermolysin on the substrate concentration. The enzymes concentration was 2.5 nM. Values are represented as mean ± SD of two independent experiments with triplicates. Solid lines, fit of the data to the Michaelis-Menten equation (R^2^ = 0.9939 for protealysin and R^2^ = 0.9755 for thermolysin).

The dependence of the initial rates of Abz-RSVIK(Dnp) hydrolysis by PLN and TLN on the substrate concentration fits the Michaelis-Menten equation (Fig. 3). According to the kinetic parameters, the efficiency (k_cat_/K_M_) of substrate hydrolysis by PLN is roughly three times higher (Table 1), which can be attributed to the changes in k_cat_ rather than in K_M_. Thus, the substrate binds equally well to both enzymes, and the decreased efficiency of TLN can be due to inaccurate positioning of the bond to be digested relative to the catalytic site as a result of too deep entrance of the small lateral radical at P1′ of the substrate’s Val into the deep S1′ pocket of TLN^27^.

**Table 1:** Kinetic parameters for hydrolysis of Abz-RSVIK(Dnp) by protealysin and thermolysin at 37 °C.

Enzyme	K_M_ ± SE, μM	k_cat_ ± SE, s^−1^	k_cat_/K_M_ ± SE, s^−1^μM^−1^
Protealysin	35 ± 4	21 ± 1	0.60 ± 0.07
Thermolysin	33 ± 8	7 ± 1	0.21 ± 0.06
PLN/TLN	1.1	3.0	2.8

SE, standard error; PLN, protealysin; TLN, thermolysin.

This result critically differs from the above-mentioned pattern of FAGLA hydrolysis where the efficiency of TLN is 100 that of PLN^12^. Poor efficiency of PLN as a substrate can be attributed to the Leu-P1′ inconformity to the PLN’s substrate specificity. However, the hydrolysis of protein substrates at sites preceding Leu has been demonstrated for PLN and its close homolog ECP32/grimelisin^29,35^, which suggests that amino acids at non-P1′ positions are more significant for the PLN-substrate interaction. This agrees with the previously proposed hypothesis that the efficiency of substrate binding depends on the amino acid at the substrate P2′ position more for PLN than for TLN based on the analysis of 3D structures^27^.

Thus, Abz-RSVIK(Dnp) is the first low-molecular-weight substrate efficiently hydrolyzed by PLN and can be used to study its catalytic properties. In addition, the substrate can be used to assay activities of other M4 peptidases.

### Hydrolysis of the substrate by other enzymes

The amino acid sequence of Abz-RSVIK(Dnp) suggests that this substrate can be hydrolyzed by both M4 peptidases and other proteases. We tested this suggestion using several proteases whose specificity (according to published data) allows the cleavage of the proposed substrate. We used trypsin with the preference for peptide bonds next to Arg and Lys; сhymotrypsin cleaving bonds next to Val, Ile, and Ser^36^; and savinase cleaving bonds next to Val, Ile, and Arg^37^ (however, these amino acids are not preferred at P1 for chymotrypsin and savinase); as well as pronase E, a mixture of *Streptomyces griseus* peptidases with different activity and specificity^38^.

**Figure 4:**
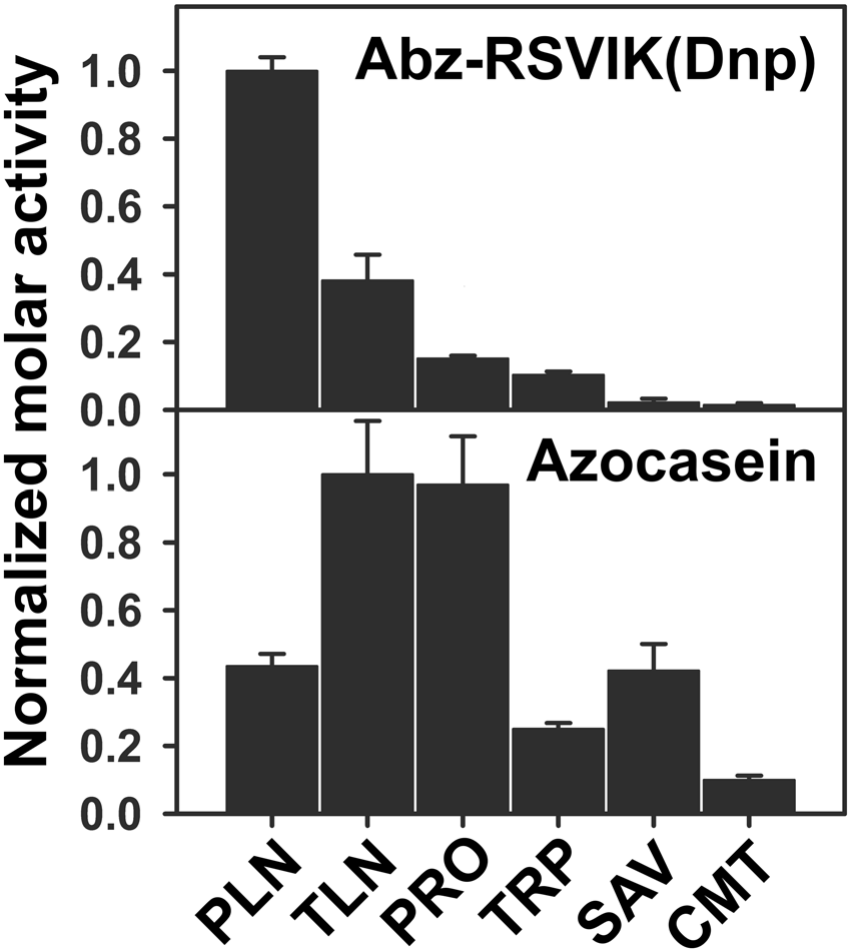
Hydrolysis of Abz-RSVI-K(Dnp) and azocasein by different enzymes. PLN, protealysin; TLN, thermolysin; PRO, pronase E; TRP, trypsin; SAV, savinase; and CMT, chymotrypsin. The activity was evaluated for 60 µM Abz-RSVI-K(Dnp); 2.5 nM PLN, TLN, TRP, SAV, and CMT; and 1.7 nM PRO. Enzyme concentrations were determined after Bradford. The following molecular weights were used in the calculation: PLN, 31.9 kDa; TLN, 34.6 kDa; PRO, 40 kDa (the mean molecular weight of the PRO enzymes); TRP, 23.8 kDa; SAV, 26.7 kDa; and CMT, 25 kDa. Values are represented as the mean and SD of three independent experiments.

**Figure 5:**
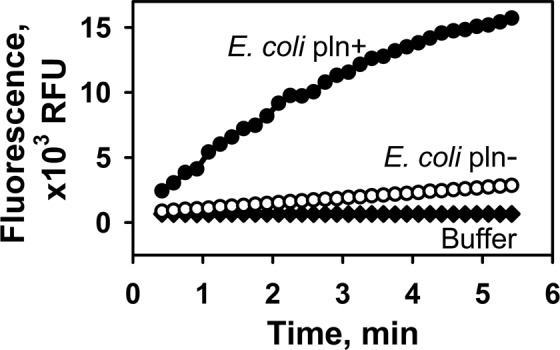
Effect of bacterial lysates on Abz-RSVI-K(Dnp). Abz-RSVI-K(Dnp) (40 µM) was incubated with: *E*. *coli pln*+, lysate of *E*. *coli* TG1 cells transformed with pSP1.8 containing the locus of *Serratia proteamaculans* genomic DNA with the protealysin gene^12^; *E*. *coli pln-*, lysate of *E*. *coli* TG1 cells transformed with the empty cloning vector pUC19; *Buffer*, buffer without cell lysates. Lysates obtained from 5∙10^4^ *E*. *coli* cells were used for the activity assays. RFU, relative fluorescence units.

This comparison demonstrates the highest efficiency of Abz-RSVIK(Dnp) hydrolysis by PLN among the tested enzymes. A parallel test using azocasein demonstrated a different pattern (Fig. 4). Despite its wide substrate specificity, PLN is not a leader here: it is inferior to TLN and pronase E and on par with savinase. The data obtained indicate that Abz-RSVIK(Dnp) is not a strictly specific substrate for PLN but its structure fits more the specificity of this metalloprotease compared to other tested enzymes (including the related TLN), which makes it acceptable considering the wide substrate specificity of PLN. At the same time, Abz-RSVIK(Dnp) is not specific enough to detect protealysin-like proteases in natural producers. This is indicated by our comparison of cell lysate activities in *E*. *coli* TG1 (pUC19) lacking M4 peptidase genes (according to the genome analysis) and the same strain transformed by the pSP1.8 plasmid obtained and described by us previously. This construct contains a fragment of *S*. *proteamaculans* DNA (~3 kb) inserted into the *Bam*HI site of pUC19. The cloned fragment contains the PLN gene with its own regulatory elements providing for its expression^12^. In the case of *E*. *coli* TG1 (pSP1.8) the activity was ~100 higher relative to *E*. *coli* TG1 (pUC19) (Fig. 5). However, the cloning vector used, pUC19, is a high-copy-number plasmid maintained at 500–700 copies per cell^39^. Thus, the PLN gene dose was by more than two orders of magnitude higher in our experiment than in the natural producer. Accordingly, one can expect low levels of this enzyme and sub-background levels of its activity in the natural producer.

## Conclusions

The first substrate applicable to quantitative studies of protealysin catalytic properties has been constructed. This substrate can also be used for activity assays of other M4 peptidases.

## Methods

### Materials

In this work we used tris-(hydroxymethyl)aminomethane (Tris) (Amresco, USA), ammonium bicarbonate, azocasein (Sigma, USA), dimethyl sulfoxide (DMSO) (MP Biomedicals, France), thermolysin (TLN) (Serva, Germany), pronase E (Serva, Germany), savinase (Novozymes, Denmark), trypsin (Spofa, Czech Republic), and chymotrypsin (Koch Light Laboratories, UK).

An internally quenched fluorescent peptide substrate for protealysin, 2-aminobenzoyl-L-arginyl-L-seryl-L-valyl-L-isoleucyl-L-(ε-2,4-dinitrophenyl)lysine (Abz-RSVIK(Dnp)), was synthesized by Peptide 2.0 (Chantilly, USA). The peptide purity was over 98% as determined by HPLC analysis. The results of the chromatography and mass-spectrometry analysis of Abz-RSVIK(Dnp) are given in the Supplementary Information File.

His_6_-tagged protealysin (PLN) was purified as described elsewhere^29^. Briefly, *E*. *coli* cells BL-21 (DE3) [pProPlnHis_6_] were grown with aeration at 37 °C in Lennox LB^40^ containing 100 μg/ml ampicillin. PLN synthesis was induced by 0.5 mM of isopropyl-β-D-thiogalactopyranoside (Sigma, USA) using standard protocol. Four h after induction, cells were collected by centrifugation, resuspended in 50 mM Tris-HCl (pH 8.0), and ultrasonicated. After centrifugation, the supernatant was applied onto a Ni-NTA XPure Agarose Resin (UBPBio, USA) column equilibrated with the same buffer. PLN was eluted with 100 mM imidazole. The fractions containing the target protein were concentrated on an Amicon Ultra-15 centrifugal filter unit (Millipore, USA) applied onto a Superdex 75 HR 10/30 column (GE Healthcare, Sweden) and eluted with the same buffer containing 0.15 M NaCl. Finally, PLN was dialyzed against 50 mM NH_4_HCO_3_ and lyophilized.

All other reagents were of reagent grade and purchased from Dia-M (Russia).

### Bioinformatics

Sequences of PLPs were extracted by protein BLAST (http://blast.ncbi.nlm.nih.gov) from the database of non-redundant protein sequences (nr) using the PLN precursor sequence (GenBank ID: AAV88082.1) as a query. Sequences whose identity to other sequences exceeded 85% were excluded using the CD-HIT web server (http://www.bioinformatics.org/cd-hit)^41^. The multiple sequence alignment of the remaining sequences was constructed by Clustal X2 (http://www.clustal.org/clustal2)^42^. The alignment was manually inspected to remove the sequences lacking the PPL motif. The resulting set included 821 sequences of PLPs. The LOGO presentation of the consensus sequence was generated by WebLogo 3.6 (http://weblogo.threeplusone.com)^43^. The sequences used for LOGO generation are presented in the Supplementary Sequences File.

### Protein and Abz-RSVIK(Dnp) concentrations

The concentrations of PLN and TLN were determined spectrophotometrically at 280 nm using the extinction coefficients ε_280nm_ = 52370 M^−1^ cm^−1^ and ε_280nm_ = 58200 M^−1^ cm^−1^, respectively. The extinction coefficients were calculated from the amino acid sequence using the ProtParam tool (http://www.expasy.org/tools/protparam.html).

The concentrations of other proteins were assayed after Bradford^44^ with modifications^45^ using IgG as a standard.

The Abz-RSVIK(Dnp) concentration was determined by the absorption of 2,4-dinitrophenyl at 365 nm using the extinction coefficient ε_365nm_ = 17300 M^−1^ cm^−1 ^^46^. The optical density was recorded 10–15 s after the solution was introduced into the optical cell.

The optical density was registered using an Agilent 8453 UV-Vis Spectrophotometer.

### Solubility of Abz-RSVIK(Dnp)

The stock solutions (6 µl) in DMSO were supplemented with 500 µl of 50 mM Tris-HCl (pH 7.4). The solutions were incubated at room temperature and the concentration changes were controlled spectrophotometrically for 24 h (Supplementary Fig. S3).

### Identification of the cleavage site of the substrate

The cleavage site was identified in 10 mM NH_4_HCO_3_. The volume of the reaction mixture was 100 µl with 20 µM Abz-RSVIK(Dnp), 2.5 nM PLN, and 7.5 nM TLN. The mixture was incubated for 0, 20, or 40 min at 37 °C and immediately frozen at −20 °C and lyophilized. Control samples were produced in a similar way but without the enzyme or the substrate. Dry samples were dissolved in 150 µM methanol (HPLC grade, ≥99.9%; Biosolve, Netherlands). The hydrolysis products were analyzed using an amaZon SL mass spectrometer (Bruker Daltonics, Germany) in positive ion mode. The samples were introduced directly into the ionization chamber with a flow rate of 200 µl/h. Electrospray ionization was performed at 4000/500 V and the drying gas (nitrogen) temperature of 160 °C. The ion charge control value (ICC) was set to 200 000 with a maximum accumulation time of 50 ms. The mass measurement range in the analyzer was 100–1800 m/z. The raw data were processed with Data Analysis 4.0 (Bruker Daltoniks, Germany).

### Protease activity assay using Abz-RSVIK(Dnp) and hydrolysis kinetic parameters

Desired substrate concentrations were obtained by dilution of the stock in DMSO in 50 mM Tris-HCl (pH 7.4). In the activity assay, DMSO concentration did not exceed 2% in the reaction mix.

For the activity assay, 100 µl of the substrate solution at 2–130 mM in 50 mM Tris-HCl (pH 7.4) was added into a well of a black 96-well plate. After incubation at 37 °C for 10 min, 25 µl of the substrate were taken away and replaced with 25 µl of the enzyme solution in the same buffer preheated at 37 °C, and time-related fluorescence changes were monitored on an Infinite M200 Pro (Tecan, Switzerland). Measurements were taken at 37 °C every 10 s for 2–5 min at the excitation and emission wavelengths of 320 and 420 nm, respectively. Fluorescence varied linearly for 3 min. The initial hydrolysis rates were determined from the data obtained within the first 95 s of the reaction. The concentrations of PLN and TLN in the reaction mix were 2.5 nM. In parallel with the hydrolysis rate measurements, the control was conducted with the buffer added to a well instead of an enzyme solution. The main goal of this control was to determine the substrate concentration in the reaction mix. After the incubation, the substrate solution was transferred into a spectrophotometer cell to measure its optical density at 365 nm. The concentration calculated from the obtained OD was taken as the initial substrate concentration in the hydrolysis reaction.

A calibration curve was plotted to convert the obtained relative fluorescence data into the hydrolyzed substrate concentration. Abz-RSVIK(Dnp) was completely hydrolyzed by PLN after incubation of the reaction mix containing 15 µM substrate and 12.5 nM PLN in 50 mM Tris-HCl (pH 7.4) at 37 °C for 15 min. The course of the reaction was followed by fluorescence changes as described above. The hydrolysis was considered as complete when the fluorescence signal remained steady even after an extra portion of the enzyme was added (Supplementary Fig. S4). After the incubation, serial dilutions of the reaction mix were made, introduced into wells of a 96-well plate and fluorescence was measured as described above. 2,4-Dinitrophenyl was quantified spectrophotometrically in each well as described above. The resulting values were used to plot a calibration curve in coordinates of relative fluorescence units, μM.

PLN and TLN remained active under the experimental conditions for 1 h (Supplementary Fig. S5).

The experimental data were fit to the Michaelis-Menten equation to calculate the K_M_ и k_cat_. Nonlinear regression was performed using SigmaPlot 11 (Systat Software, USA).

### Protease activity assay using azocasein

A mixture of 100 µl 1% azocasein in 50 mM Tris-HCl (pH 7.4) with 50 µl of the enzyme in the same buffer was incubated at 37 °C for 1 h. The reaction was stopped by adding 200 µl of 10% trichloroacetic acid. After centrifugation (12,400 g for 10 min), 250 µl of the supernatant was mixed with 50 µl of 4 M NaOH to measure the absorbance at 450 nm on an Infinite M200 Pro (Tecan, Switzerland). The activity unit was defined as the amount of enzyme that changed the absorbance by one optical density unit per min.

### *Escherichia coli* cultivation and sampling

*E*. *coli* TG1 cells transformed with pUC19 or pSP1.8^12^ were grown overnight with aeration at 37 °C in Lennox LB^40^ containing 100 μg/ml ampicillin. After measuring the culture optical densities at 600 nm (OD_600nm_), 6 ml of the cultures were centrifuged at 3200 g for 10 min. The cell pellets were resuspended in 0.6 ml of 50 mM Tris-HCl (pH 7.4) and ultrasonicated at 4 °C for 60 s with 30% of the duty cycle. After centrifugation (8600 *g*, 10 min, 4°С), the supernatants were diluted with the same buffer. One ml of the final solution contained lysate made from ~2∙10^6^ bacterial cells. Lysed cells were quantified at OD_600_ considering that OD_600nm_ = 1 corresponds to 8∙10^8^ cells/ml^47^. For the protease activity assay using Abz-RSVIK(Dnp), 25 µl of each diluted solution was used.

## Supplementary information

Supplementary information

Supllementary sequences

## Data Availability

All data generated or analyzed during this study are included in this published article (and its Supplementary Information Files).
