# Supplementary material for: An Internally Quenched Fluorescent Peptide Substrate for Protealysin
Source: Sci Rep. 2019 Oct 4;9:14352. doi: 10.1038/s41598-019-50764-2 (PMC6778150; doi:10.1038/s41598-019-50764-2)
Supplement: Supplementary file 1 — Supplementary information [file 41598_2019_50764_MOESM1_ESM.pdf]

SUPPLEMENTARY INFORMATION

An Internally Quenched Fluorescent Peptide Substrate for Protealysin

Maria A. Karaseva[1], Ksenia N. Chukhontseva[1], Irina S. Lemeskina[1], Marina L. Pridatchenko[2], Sergey V. Kostrov[1], and Ilya V. Demidyuk[1]

[1]Institute of Molecular Genetics, Russian Academy of Sciences, Moscow, Russia
[2]V.L. Talrose Institute for Energy Problems of Chemical Physics, N.N. Semenov Federal Research Center of Chemical Physics, Russian Academy of Sciences, Moscow, Russia

Correspondence and requests for materials should be addressed to I.V.D. (email: duk@img.ras.ru)

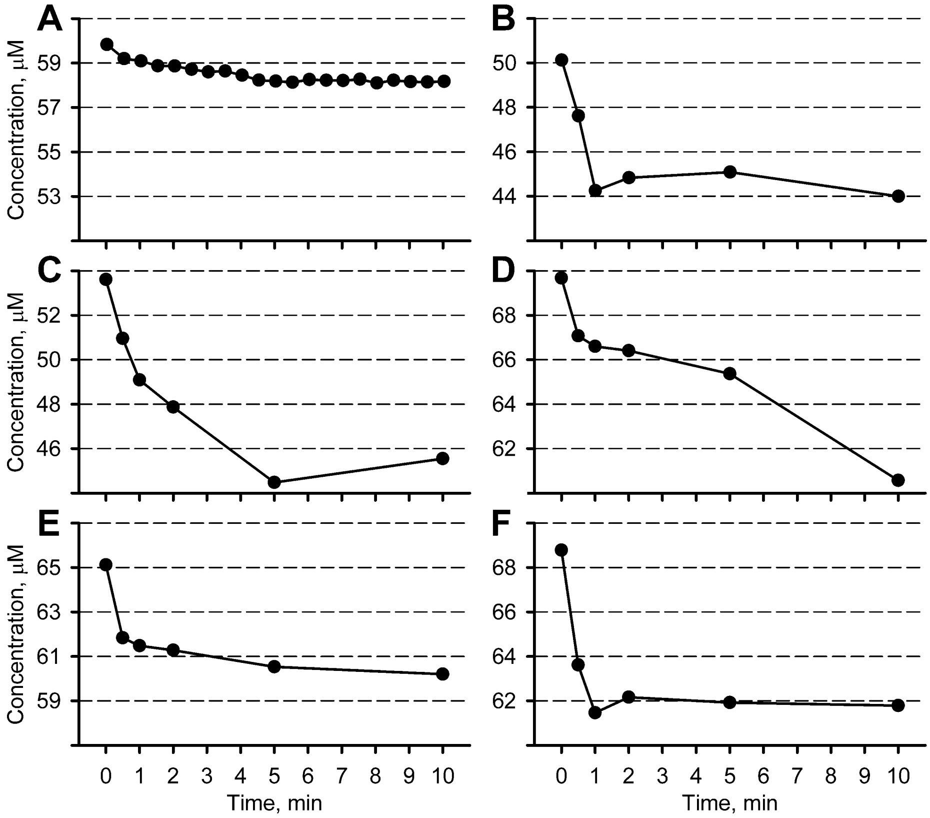

Figure S1. Abz-RSVIK(Dnp) sorption. A. Changes in Abz-RSVIK(Dnp) concentration during incubation in a quartz cell for spectrophotometry. B. Changes in Abz-RSVIK(Dnp) concentration during incubation in a microplate for fluorescence-based assays (Costar, Cat. #3915). C, D, E, and F. Changes in Abz-RSVIK(Dnp) concentration during incubation in microcentrifuge tubes from different manufacturers: SSI (Cat. #1260), Eppendorf (Protein LoBind, Cat. #022431081), GenFollower (Cat. #MCTB015), and Corning-Costar (Cat. #3620), respectively.

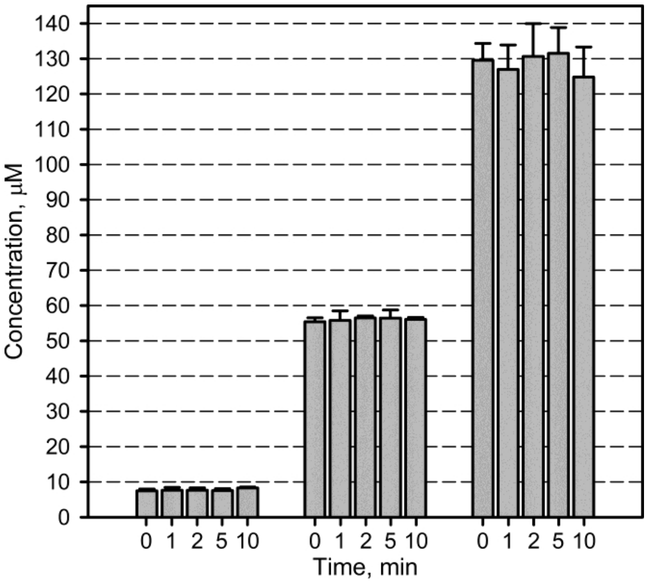

Figure S2. Abz-RSVIK(Dnp) concentration remains constant during incubation in pretreated microplates for fluorescence-based assays. Such pretreatment of plates was used in further enzyme activity assays. Different substrate concentrations (100 μl) were added to wells of a 96-well microplate. After incubation at 37°C for 10 min, 25 μl were discarded and 25 μl of 50 mM Tris-HCl (pH 7.4) was mixed with the well contents. The substrate concentration was quantified spectrophotometrically immediately after the buffer was added as well as after time intervals specified. Values are represented as the mean and SD of three independent experiments.

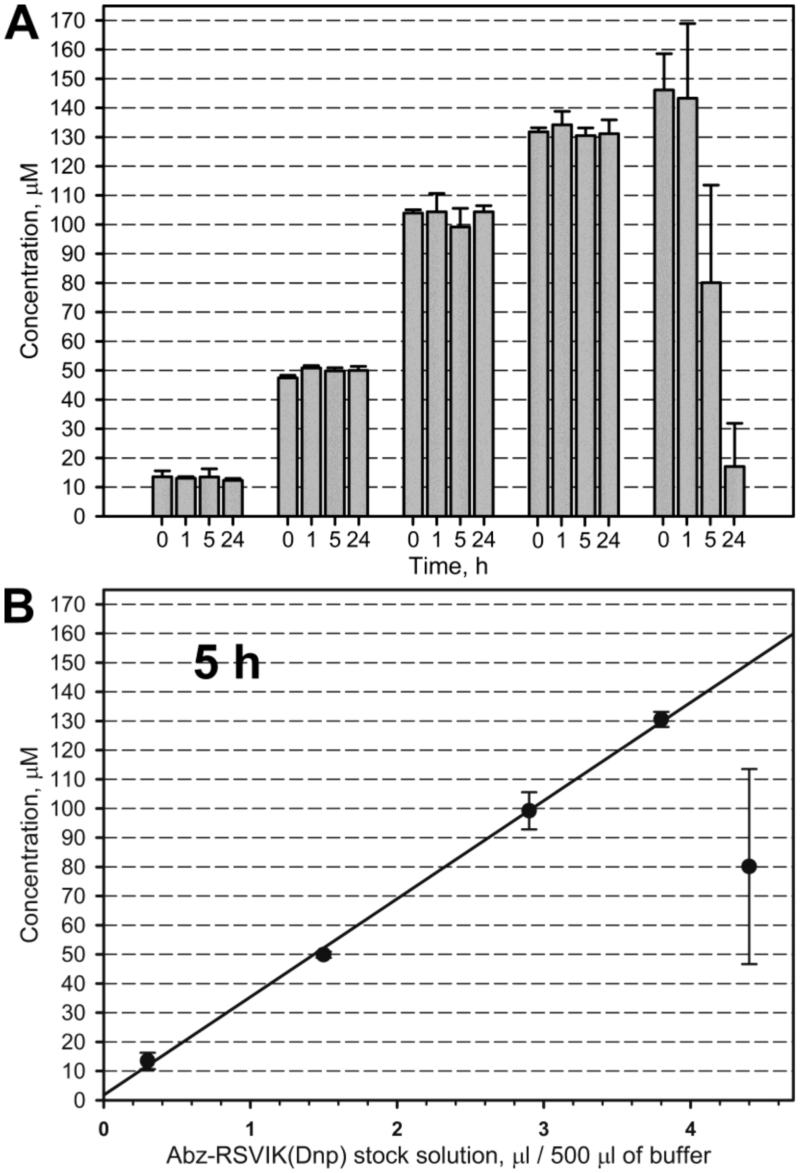

Figure S3. Solubility of Abz-RSVIK(Dnp). Different substrate concentrations were prepared in microcentrifuge tubes (SSI). The stock solution was diluted with DMSO 1.3-20 times. The resulting solutions (6 µl) were supplemented with 500 µl of 50 mM Tris-HCl (pH 7.4). The solutions were incubated at room temperature and the concentration changes were controlled spectrophotometrically at 365 nm. The first measurement was made after 10 min ("0 h") and then after time periods specified here. Values are represented as the mean and SD of three independent experiments. A. Measured substrate concentrations for all dilutions and time periods. B. Relationship between substrate concentration and stock quantity 5 h after dilution.

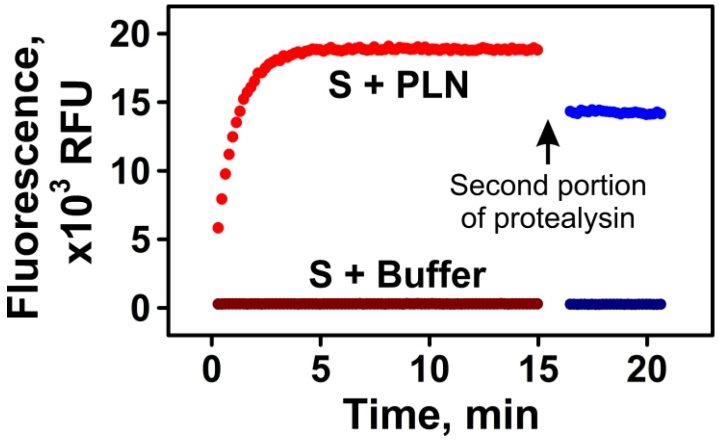

Figure S4. Complete hydrolysis of Abz-RSVIK(Dnp). Substrate solution (100 µl) in was added to a well of a 96-well plate and incubated at 37°C for 10 min. After the incubation, 25 µl of substrate were discarded and 25 µl of 50 nM protealysin (PLN) were added. The reaction mixture was incubated at 37°C for 15 min and the fluorescence signal was monitored. Then, 25 µl of the mixture were replaced with 25 µl of 50 nM PLN and the fluorescence signal was monitored during the incubation at 37°C for 5 min. The interval between the measurements was 1 min 30 s. After the first and second addition of the enzyme, the reaction mixture contained 15 µM Abz-RSVIK(Dnp) + 12,5 nM PLN and 11.25 µM Abz-RSVIK(Dnp) + 21.9 nM PLN, respectively. The reaction was carried out in 50 mM Трис-HCl (pH 7.4). Red and blue circles indicate the fluorescence signal after the first and second addition of PLN, respectively (S + PLN); while brown and dark blue circles indicate the control fluorescence where 25 µl of the buffer was added instead of PLN (S + Buffer).

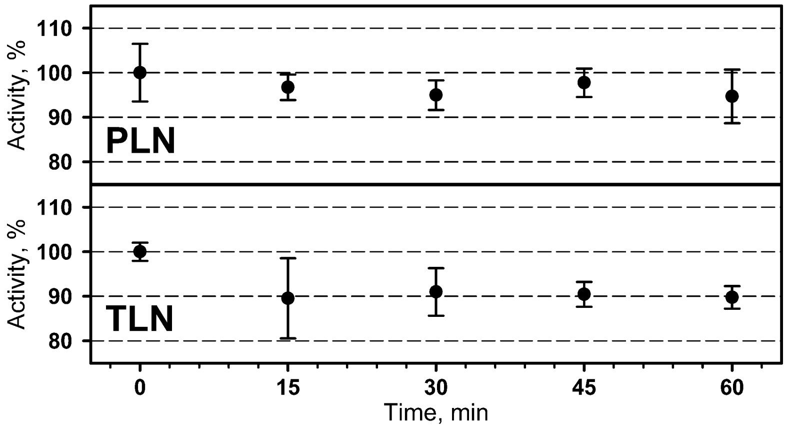

Figure S5. Experimental stability of protealysin (PLN) and thermolysin (TLN). Enzymes (10 nM) were incubated at 37°C for 15, 30, 45, or 60 min and placed on ice. Enzyme activities were assayed using Abz-RSVIK(Dnp) as described in the Methods section. The concentration of the enzymes in the reaction mixture was 2.5 nM. The substrate concentration was 15 and 13 µM for PLN and TLN, respectively. The values are represented as the mean and SD of three measurements.

THE RESULTS OF THE CHROMATOGRAPHY AND MASS-SPECTROMETRY ANALYSIS OF Abz-RSVIK(Dnp) BY PEPTIDE 2.0

Data Analysis Report

**Peptide Name**	1
**Sequence**	(2-Abz)-RSVIK(Dnp)
**M.W. (Theoretical)**	886.86 g/mol
**Reference No.**	132537-001
**% of Hydrophobic amino acids**	40%
**% of Acidic amino acids**	0%
**% of Basic amino acids**	40%
**% of Neutral amino acids**	20%

Test	Results
**Appearance**	Lyophilized material
**Amount**	50.0 mg
**MS ($M+H^+$)**	887.14<br>(see raw data enclosed)
**MS ($M+Na^+$)**	
**MS ($M+K^+$)**	
**Purity (HPLC)**	98.66% (HPLC, 220 nm, C18, linear gradient)<br>(see raw data enclosed)
**Storage Conditions**	-20 °C
**Remarks**	This product is supplied as trifluoroacetate salt

Use recommended within 6 months from manufacturing date

(Analytical Data see next pages)

HPLC Data

HPLC Column	Agela (250×4.6mm I.D.) C18	
Detection wavelength	220 nm	
Gradient	38-54%B in 16 min	
Buffer A	0.05%TFA in H_2O	
Buffer B	0.05%TFA in 90% CH_3CN	
Gradient	A	B
0.01 min	62%	38%
16.0 min	46%	54%
16.1 min	0%	100%
26.0 min	STOP	

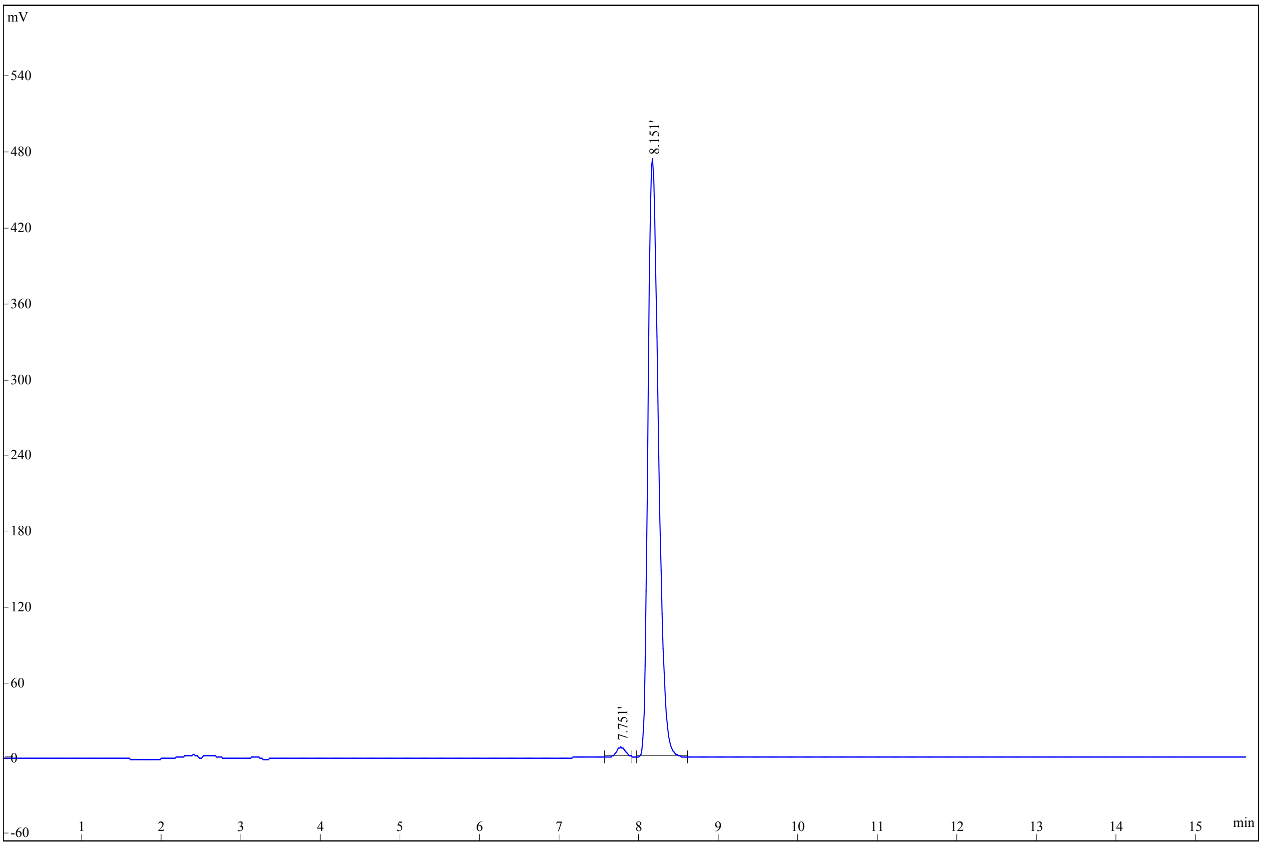

Peak Results

Rank	Time	Conc.	Area	Height
1	7.751	1.347	59079	7591
2	8.151	98.66	4327588	475139
Total		100	4386667	482730

MS Data

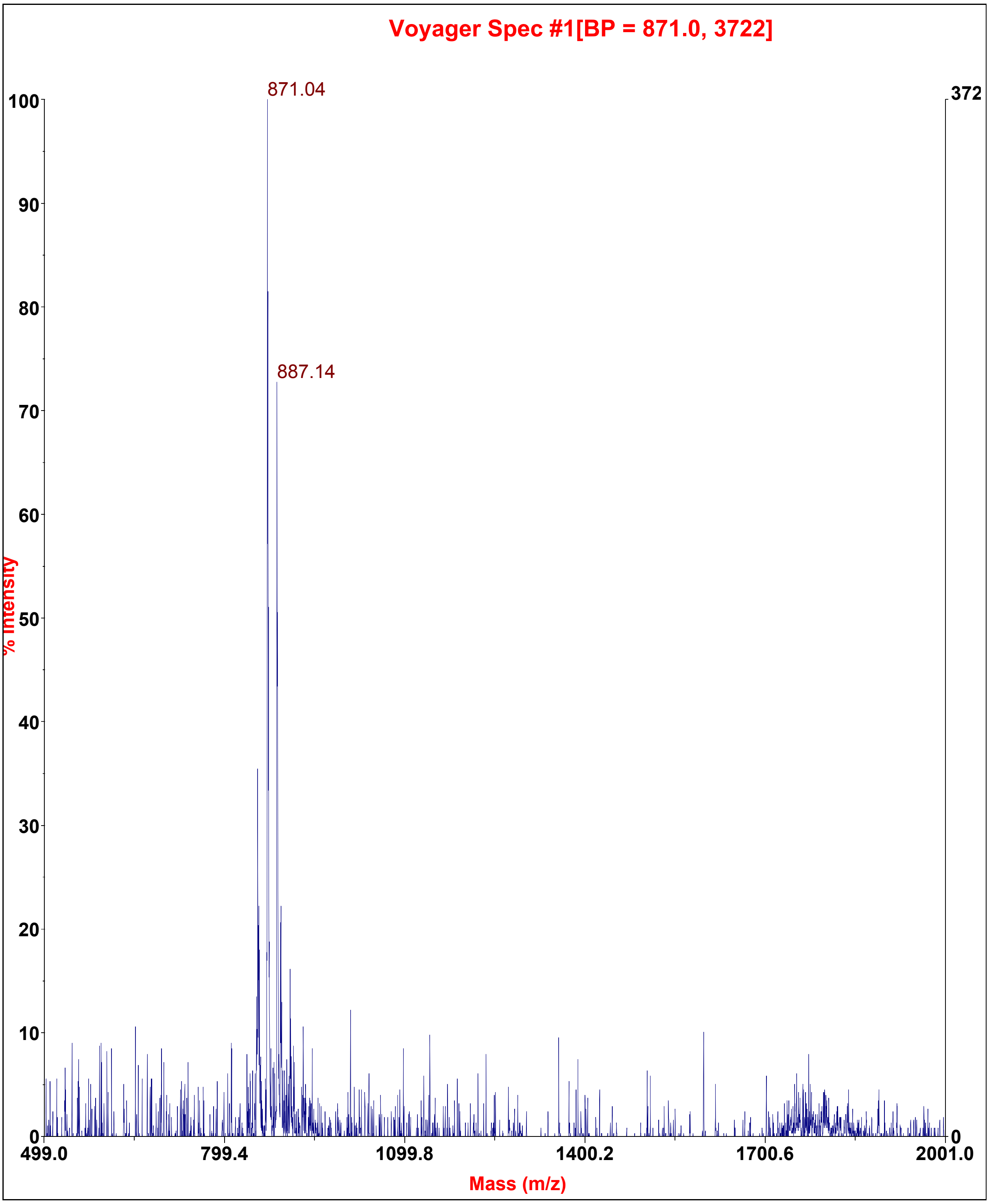
Voyager Spec #1[BP = 871.0, 3722]
871.04
887.14
% Intensity
100
90
80
70
60
50
40
30
20
10
0
372
0
499.0
799.4
1099.8
1400.2
1700.6
2001.0
Mass (m/z)
