# Supplementary material for: An Internally Quenched Fluorescent Peptide Substrate for Protealysin
Source: Sci Rep. 2019 Oct 4;9:14352. doi: 10.1038/s41598-019-50764-2 (PMC6778150; doi:10.1038/s41598-019-50764-2)
Supplement: Supplementary file 2 — Supllementary sequences [file 41598_2019_50764_MOESM2_ESM.zip › SUPPLEMENTARY TITLE.pdf]

SUPPLEMENTARY INFORMATION

An Internally Quenched Fluorescent Peptide Substrate for Protealysin

Maria A. Karaseva[1], Ksenia N. Chukhontseva[1], Irina S. Lemeskina[1], Marina L. Pridatchenko[2], Sergey V. Kostrov[1], and Ilya V. Demidyuk[1]

[1]Institute of Molecular Genetics, Russian Academy of Sciences, Moscow, Russia.
[2]Talrose Institute for Energy Problems of Chemical Physics, Russian Academy of Sciences, Moscow, Russia.

Correspondence and requests for materials should be addressed to I.V.D. (email: duk@img.ras.ru)
